# Cetuximab plus platinum-based chemotherapy in head and neck squamous cell carcinoma: a randomized, double-blind safety study comparing cetuximab produced from two manufacturing processes using the EXTREME study regimen

**DOI:** 10.1186/s12885-016-2064-0

**Published:** 2016-01-14

**Authors:** Denis Soulières, Jose Luis Aguilar, Eric Chen, Krzysztof Misiukiewicz, Scott Ernst, Hyun Jung Lee, Katherine Bryant, Shuang He, Coleman K. Obasaju, Shao-Chun Chang, Steve Chin, Douglas Adkins

**Affiliations:** Centre Hospitalier de l’Université de Montréal, Montréal, Québec Canada; Instituto Nacional de Cancerologia, Mexico City, Mexico; Princess Margaret Hospital, Toronto, Ontario Canada; Mount Sinai School of Medicine Tisch Cancer Institute, New York, NY USA; London Regional Cancer Center, London, Ontario Canada; Eli Lilly and Company, Indianapolis, IN USA; Washington University School of Medicine, 660 S. Euclid, Box 8056, St. Louis, MO 63110 USA

**Keywords:** Carcinoma, squamous cell, Cetuximab, Head and neck cancer, Safety

## Abstract

**Background:**

Cetuximab, in combination with platinum chemotherapy plus 5-fluoruracil (5-FU), is approved for the first-line treatment of recurrent/metastatic squamous cell carcinoma of the head and neck (SCCHN). Cetuximab manufactured by ImClone (US commercial cetuximab) potentially results in higher systemic exposures than cetuximab manufactured by Boehringer Ingelheim (BI-manufactured cetuximab). This prospective, randomized, double-blind study compared the safety profiles of the two cetuximab formulations.

**Methods:**

Patients with previously untreated locoregionally recurrent and/or metastatic SCCHN were randomly assigned to receive the same dose of US commercial cetuximab (Arm A) or BI-manufactured cetuximab (Arm B), each in combination with cisplatin or carboplatin plus 5-FU. The primary outcome was all-grade, all-cause treatment-emergent adverse events (TEAEs).

**Results:**

The majority of patients experienced ≥1 TEAE, regardless of causality (Arm A: 75/77 patients, 97.4 %; Arm B: 68/71 patients, 95.8 %). TEAEs with the highest incidence included nausea, fatigue, and hypomagnesemia in both arms. The absolute risk difference between the two arms for patients experiencing at least one adverse event (AE) was 0.029 (*p* = 0.281, 95 % confidence interval [CI]: -0.024, 0.082) for AEs regardless of causality and 0.005 (*p* = 0.915, 95 % CI: -0.092, 0.103) for AEs possibly related to study drug. There were no significant differences between the two arms in the incidence of acneiform rash, cardiac events, infusion reactions, or hypomagnesemia. Overall survival, progression-free survival, and overall response rates were similar in the two arms.

**Conclusions:**

There were no clinically meaningful differences in safety between US commercial cetuximab and BI-manufactured cetuximab in combination with platinum-based therapy with 5-FU in patients with locoregionally recurrent and/or metastatic SCCHN. The use of US commercial cetuximab in this combination chemotherapy regimen did not result in any unexpected safety signals. The efficacy results of this study are consistent with the efficacy results of the cetuximab arm of the EXTREME study.

**Trial registration:**

ClinicalTrials.gov NCT01081041; date of registration: March 3, 2010).

## Background

Head and neck cancer is the sixth most common cancer worldwide, with more than 650,000 new cases diagnosed each year [1]. Treatment options for patients with recurrent/metastatic squamous cell carcinoma of the head and neck (SCCHN) are limited. Currently, the standard of care (category 1 evidence) for recurrent/metastatic SCCHN is the regimen used in the EXTREME study (Erbitux in First-Line Treatment of Recurrent or Metastatic Head and Neck Cancer), consisting of cetuximab in combination with cisplatin or carboplatin plus 5-fluorouracil (5-FU) [2, 3]. Historically, median survival with chemotherapy is approximately 6 months and the 1-year survival rate is approximately 20 % [2]. The addition of cetuximab, an anti-epidermal growth factor receptor (EGFR) monoclonal antibody, to cytotoxic agents has shown a significant increase in response rate [3, 4] and a significant survival benefit [3] in recurrent/metastatic SCCHN.

In the EXTREME study, conducted in 17 European countries, 442 patients with previously untreated recurrent/metastatic SCCHN were randomized to receive chemotherapy alone (cisplatin or carboplatin plus 5-FU) or in combination with cetuximab [3]. There were statistically significant and clinically meaningful improvements in all efficacy endpoints (overall survival [OS], progression-free survival [PFS], and overall response rate [ORR]) in the cetuximab plus chemotherapy group compared with the chemotherapy-alone group. Overall, the safety data from the EXTREME study indicated that the addition of cetuximab to chemotherapy did not affect tolerability and that the adverse event (AE) profile for this cetuximab-chemotherapy regimen was consistent with that expected for the agents used [3]. Adverse events of interest for cetuximab include acneiform rash, cardiac events, infusion reactions, and hypomagnesemia [3, 5]. Based on the results of the EXTREME study, cetuximab was approved by the European Union (EU) for the treatment of patients with SCCHN in combination with platinum-based chemotherapy for recurrent and/or metastatic disease [6], and later by the United States (US) Food and Drug Administration (FDA) for the first-line treatment of patients with recurrent locoregional disease or metastatic SCCHN in combination with platinum-based therapy with 5-FU [5].

The cetuximab clinical supply for the EXTREME study was manufactured by the Europe-based company Boehringer Ingelheim (BI-manufactured cetuximab). Population pharmacokinetic data indicate that cetuximab manufactured by the US-based company ImClone (US commercial cetuximab) is associated with approximately 22 % higher systemic drug exposure relative to BI-manufactured cetuximab, due to decreased clearance [5]. Due to the potential for increased exposure and the possibility of a greater incidence and severity of adverse reactions with US commercial cetuximab compared with BI-manufactured cetuximab [5], a prospective study of the two cetuximab formulations was conducted. This study compared the safety profiles of US commercial cetuximab and BI-manufactured cetuximab, each in combination with cisplatin or carboplatin plus 5-FU (the regimen used in the EXTREME study [3]), in patients with locoregionally recurrent and/or metastatic SCCHN.

## Methods

### Study population

The patient eligibility criteria were similar to those of the EXTREME study [3]. Patients with histologically or cytologically confirmed locoregionally recurrent and/or metastatic SCCHN not suitable for local therapy were eligible for this study. Other eligibility criteria included: age ≥18 years; measurable or evaluable disease, as defined by Response Evaluation Criteria in Solid Tumors (RECIST) version 1.0; Karnofsky Performance Status (KPS) score of at least 70; and adequate hematologic, renal, and hepatic function. Patients were excluded from the study if they had: previous systemic chemotherapy, unless required as part of multimodal treatment for locally advanced head and neck cancer that was completed more than 4 months before study entry; previous treatment with monoclonal antibody therapy, other signal transduction inhibitors, or EGFR targeting therapy, except for previous cetuximab treatment given as part of a multimodal treatment for locally advanced head and neck cancer that was completed more than 4 months before study entry; nasopharyngeal carcinoma; or other concomitant anticancer therapies.

The study was conducted in hospital-based clinics in North America. The study protocol was approved by the ethics review board at each site (see below) and was conducted in accordance with Good Clinical Practice guidelines and the Declaration of Helsinki. All patients provided written informed consent before undergoing any study procedure. The study was registered at www.clinicaltrials.gov (NCT01081041) [7].

### Ethics review boards

The following ethics review boards approved the study protocol: US Oncology, Western Institutional Review Board, Washington University Medical Center, Dallas VA Medical Center IRB, Committee for the Protection of Human Subjects, Wayne State University, Wake Forest University School of Medicine, Cleveland Clinic of Weston Florida, University of Illinois College of Medicine, Scott & White Institutional Review Board, Decatur Memorial Hospital, Medical University of South Carolina, Stratton VA Medical Center, Veterans Affairs Medical Center, Medical College of Georgia, Translational Research in Oncology-US Inc, Mount Sinai School of Medicine Dermatology Clinical Trials, Comite Bioetico para la Investigacion Clinica SC, Centro Anticanceroso Cruz Roja Mexicana, Instituto Nacional de Cancerologia, Centro Estatal de Cancerologia, Antiguo Hospital Civil de Guadalajara, Ontario Cancer Research Ethics Board, Alberta Health Services, and Comite D'Ethique De La Recherche.

### Study design

This was a multicenter, Phase 2 study with a single-arm, open-label, 30-patient safety lead-in phase followed by a two-arm, randomized, double-blind phase in patients with locoregionally recurrent and/or metastatic SCCHN who had not received prior systemic chemotherapy. The 30-patient lead-in phase was requested by the FDA to assess the safety of US commercial cetuximab before starting the randomized phase of the study.

Patients were randomly assigned to US commercial cetuximab plus chemotherapy (Arm A) or BI-manufactured cetuximab plus chemotherapy (Arm B) in a 1:1 ratio (first patient enrolled: 08 June 2010). Randomization was carried out using a computer-generated random sequence and a centralized interactive voice response system (IVRS). To maintain the blinding of patients and the personnel involved in patient evaluations and data collection, an unblinded third party was designated. The investigator provided the necessary information to the unblinded designee who then called the IVRS to obtain the patient’s treatment assignment.

A minimization principle was used to balance patient assignment between treatment arms, using a probability factor of 0.75, based on the following factors: primary tumor site (oral cavity/oropharynx vs other); previous chemotherapy (yes vs no); previous cetuximab treatment (yes vs no); KPS (<80 vs ≥80); intention to give cisplatin or carboplatin; measurable vs evaluable disease; and participating center.

### Treatment protocol

Study drugs were administered intravenously in 21-day cycles, with a 1-hour observation period between cetuximab and cisplatin/carboplatin, in the following order: (i) cetuximab 400 mg/m^2^ (2-hour infusion) on Day 1 of Cycle 1 and 250 mg/m^2^ (1-hour infusion) weekly thereafter; (ii) cisplatin 100 mg/m^2^ (1-hour infusion) or carboplatin area under the concentration curve (AUC) 5 mg/mL/min (1-hour infusion) on Day 1 of Cycles 1 to 6; (iii) 5-FU 1000 mg/m^2^/day as a continuous infusion on Days 1 through 4 of Cycles 1 to 6. Patients enrolled in the safety lead-in phase and patients randomized to Arm A received US commercial cetuximab; patients randomized to Arm B received BI-manufactured cetuximab. Patients received cisplatin or carboplatin at the discretion of the investigator. Dose modifications of cetuximab and chemotherapy were permitted according to protocol-specified criteria. Patients who completed six cycles of combination therapy continued to receive cetuximab monotherapy until disease progression, unacceptable toxicity, or any other withdrawal criterion was met. If clinically indicated, patients who discontinued chemotherapy prior to completing six cycles of combination therapy were permitted to continue with cetuximab until a withdrawal criterion was met. Patients who discontinued cetuximab were permitted to continue with chemotherapy up to six cycles.

### Assessments

Adverse events were assessed according to the Common Terminology Criteria for Adverse Events (CTCAE; Version 4.0) and the Medical Dictionary for Regulatory Activities (MedDRA; Version 16.0).

Tumor response was assessed using RECIST version 1.0. Tumor measurements were performed at baseline and every two cycles by computed tomography or magnetic resonance imaging; chest x-ray was acceptable for clearly defined lesions surrounded by aerated lung.

### Outcomes

The primary objective of the study was to prospectively compare the safety profiles of US commercial cetuximab and BI-manufactured cetuximab with respect to individual all-grade, all-cause treatment-emergent adverse events (TEAEs) occurring at any time during the treatment period. A TEAE was defined as an event that first appeared or an event already present that worsened in severity following the first dose exposure and up to 30 days after the last dose of study treatment. Secondary objectives included overall safety, OS, PFS, and ORR.

### Statistical analysis

Planned enrollment was approximately 230 patients: 30 patients for the safety lead-in phase and 200 patients for the two-arm, randomized, double-blind phase. The sample size was based on practical and clinical considerations to ensure that the safety profile could be appropriately compared between the two treatment arms and was not based on any statistical assumptions or hypotheses.

For the safety lead-in phase, the safety and efficacy analyses were conducted on the safety lead-in population, which consisted of all patients enrolled in this phase of the study. For the randomized phase, the safety and efficacy analyses were conducted on the randomized and treated (RT) population, which consisted of all patients in the two-arm, randomized, double-blind phase of the study who received at least one dose of any of the study drugs (cetuximab, cisplatin/carboplatin, and 5-FU).

The primary outcome was all-grade, all-cause TEAEs in the RT population. The risk difference for the occurrence of each AE was calculated and the 95 % confidence interval (CI) and *p* value were generated using normal approximation. The false discovery rate method of multiplicity adjustment was applied to control the type I error (the false positive rate) when comparing individual AEs. Adverse events of special interest were hypomagnesemia and the composite terms acneiform rash, cardiac event, and infusion reaction. Exposure to cetuximab and chemotherapy was reported as median duration plus 25^th^ and 75^th^ percentiles and relative dose intensity (actual dose delivered as a percentage of planned dose). Overall survival was defined as the time from randomization to death; PFS was defined as the time from randomization to the first objective progression of disease or death. The Kaplan-Meier method was used to estimate median OS and PFS. Log-rank and Wilcoxon statistics were calculated to provide between-treatment comparisons unadjusted for covariates for OS and PFS. The ORR was calculated as the percentage of patients with a confirmed (within 28 days) best response of complete response (CR) or partial response (PR). The disease control rate (DCR) was calculated as the percentage of patients with a confirmed best response of CR, PR, or stable disease. Analyses were performed using SAS version 9.2 (SAS Institute, Cary, NC, USA).

Because of manufacturing process changes, Boehringer Ingelheim stopped manufacturing the cetuximab formulation used in the EXTREME study. The supply of BI-manufactured cetuximab expired during the study period; upon expiration, ongoing patients in Arm B were switched to US commercial cetuximab. For the primary safety analysis, the data analysis cut-off date was the earliest date that a patient in Arm B was switched from BI-manufactured cetuximab to US commercial cetuximab (23 January 2013). For the efficacy analyses, the data analysis cut-off date was the date that the reporting database was locked for analysis (27 September 2013). For any patient in Arm B who was switched from BI-manufactured cetuximab to US commercial cetuximab, OS and PFS were censored at the time of the switch.

## Results

### Patient disposition

**Fig. 1:**
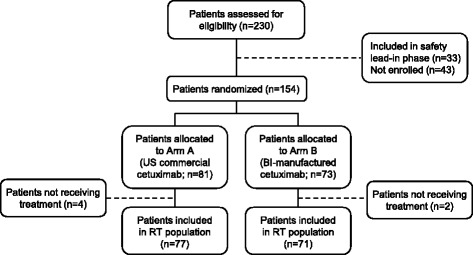
Patient flow. Abbreviations: BI = Boehringer Ingelheim; RT = randomized and treated; US = United States

A total of 33 patients were enrolled in the safety lead-in phase of the study and constituted the safety lead-in population. As the safety and efficacy results for the safety lead-in population were consistent with those for Arm A in the RT population (both of which received US commercial cetuximab), the results for this population are not presented here. In total, 81 patients were randomly assigned to US commercial cetuximab plus chemotherapy (Arm A) and 73 patients were randomly assigned to BI-manufactured cetuximab plus chemotherapy (Arm B) in the two-arm, randomized, double-blind phase of the study (Fig. 1). Of these, 77 patients in Arm A and 71 patients in Arm B received at least one dose of any study drug and constituted the RT population. In Arm B, 9/71 patients (12.7 %) switched from BI-manufactured cetuximab to US commercial cetuximab following the expiration of BI-manufactured cetuximab.

### Demographic and baseline clinical characteristics

Baseline characteristics were mostly similar between Arm A and Arm B (Table 1). The majority of patients in the RT population were male (Arm A: 68/77 patients, 88.3 %; Arm B: 55/71 patients, 77.5 %). Median age was 57.8 and 61.3 years in Arm A and Arm B, respectively. The primary tumor site was the oral cavity/oropharynx in 48/77 patients (62.3 %) in Arm A and 45/71 patients (63.4 %) in Arm B, and the larynx/hypopharynx in 18/77 patients (23.4 %) in Arm A and 13/71 patients (18.3 %) in Arm B.

**Table 1:** Patient demographics and baseline disease characteristics

Characteristic	US commercial cetuximab Arm A (N = 77)	BI-manufactured cetuximab Arm B (N = 71)
Sex, n (%)		
Male	68 (88.3)	55 (77.5)
Age, years		
Median (range)	57.8 (26.9–77.2)	61.3 (29.2–81.9)
KPS score, n (%)		
70	9 (11.7)	7 (9.9)
80	28 (36.4)	22 (31.0)
90	32 (41.6)	31 (43.7)
100	8 (10.4)	11 (15.5)
Stage of disease, n (%)		
Local	8 (10.4)	12 (16.9)
Locoregional	26 (33.8)	20 (28.2)
Metastatic	43 (55.8)	38 (53.5)
Histologic type, n (%)		
Well differentiated	3 (3.9)	6 (8.5)
Moderately differentiated	35 (45.5)	29 (40.8)
Poorly differentiated	25 (32.5)	17 (23.9)
Undifferentiated	3 (3.9)	1 (1.4)
Unable to determine	11 (14.3)	15 (21.1)
Missing/unknown	0	2 (2.8)

*Abbreviations*: *BI* Boehringer Ingelheim, *KPS* Karnofsky Performance Status, *US* United States

### Exposure to cetuximab and chemotherapy

Overall, the treatment durations of cetuximab and chemotherapy were mostly similar between Arm A and Arm B (Table 2). The relative dose intensity of cetuximab, cisplatin, and 5-FU was similar in the two arms; the relative dose intensity of carboplatin was numerically higher in Arm A than Arm B (94.3 % vs 88.4 %).

**Table 2:** Summary of treatment exposure

Treatment exposure	US commercial cetuximab Arm A (N = 77)	BI-manufactured cetuximab Arm B (N = 71)
Median duration of treatment (25^th^ percentile, 75^th^ percentile), weeks		
Cetuximab	12.4 (6.0, 18.0)	13.6 (6.0, 20.0)
Cisplatin	6.9 (3.0, 14.0)	9.0 (3.0, 18.0)
Carboplatin	12.6 (6.1, 18.0)	15.0 (7.0, 20.0)
5-FU	13.0 (6.0, 17.9)	13.0 (6.3, 20.0)
Relative dose intensity, %		
Cetuximab	87.5	87.1
Cisplatin	86.8	87.9
Carboplatin	94.3	88.4
5-FU	84.1	82.9

*Abbreviations*: *5-FU* 5-fluorouracil, *BI* Boehringer Ingelheim, *US* United States

### Safety

There were no clinically meaningful differences in safety between Arm A and Arm B, as assessed by incidence of (i) all-grade, all-cause TEAEs (the primary objective of the study), (ii) maximum CTCAE grade AEs regardless of causality, and (iii) maximum CTCAE grade AEs possibly related to study drug.

#### All-grade, all-cause TEAEs

The majority of patients in both arms experienced at least 1 TEAE, regardless of causality: 75/77 patients (97.4 %) in Arm A and 68/71 patients (95.8 %) in Arm B (Table 3). The TEAEs with the highest incidence in the two arms included nausea (Arm A: 38/77 patients, 49.4 %; Arm B: 34/71 patients, 47.9 %), fatigue (Arm A: 35/77 patients, 45.5 %; Arm B: 36/71 patients, 50.7 %), and hypomagnesemia (Arm A: 29/77 patients, 37.7 %; Arm B: 29/71 patients, 40.8 %). There were no significant differences between Arm A and Arm B in the incidence of any individual TEAE, apart from the incidence of rash, which was significantly lower in Arm A than Arm B. The TEAE of rash was reported in 10/77 patients (13.0 %) in Arm A and 19/71 patients (26.8 %) in Arm B (*p* = 0.034, 95 % CI: 0.010, 0.265).

**Table 3:** All-cause treatment-emergent adverse events occurring in ≥10 % of patients in either arm

Adverse event^a^	US commercial cetuximab Arm A (N = 77) n (%)	BI-manufactured cetuximab Arm B (N = 71) n (%)
Patients with ≥1 TEAE	75 (97.4)	68 (95.8)
Nausea	38 (49.4)	34 (47.9)
Fatigue	35 (45.5)	36 (50.7)
Hypomagnesemia	29 (37.7)	29 (40.8)
Diarrhea	25 (32.5)	22 (31.0)
Dermatitis acneiform	25 (32.5)	19 (26.8)
Vomiting	24 (31.2)	27 (38.0)
Anemia	21 (27.3)	30 (42.3)
Hypokalemia	21 (27.3)	24 (33.8)
Neutropenia	20 (26.0)	24 (33.8)
Stomatitis	20 (26.0)	21 (29.6)
Dry skin	18 (23.4)	12 (16.9)
Constipation	17 (22.1)	24 (33.8)
Mucosal inflammation	16 (20.8)	16 (22.5)
Decreased appetite	15 (19.5)	18 (25.4)
Dehydration	13 (16.9)	17 (23.9)
Neutrophil count decreased	11 (14.3)	14 (19.7)
Rash	10 (13.0)	19 (26.8)
Weight decreased	10 (13.0)	13 (18.3)
Platelet count decreased	10 (13.0)	11 (15.5)
Insomnia	9 (11.7)	11 (15.5)
Dyspnea	8 (10.4)	12 (16.9)
Thrombocytopenia	8 (10.4)	12 (16.9)
Dizziness	8 (10.4)	11 (15.5)
Paronychia	8 (10.4)	10 (14.1)
Hyponatremia	7 (9.1)	14 (19.7)
Pyrexia	7 (9.1)	8 (11.3)

^a^ Apart from rash (*p* = 0.034, 95 % CI: 0.010, 0.265), the difference between Arm A and Arm B in the incidence of all-cause TEAEs occurring in ≥10 % of patients in either arm was not statistically significant

*Abbreviations*: *BI* Boehringer Ingelheim, *TEAE* treatment-emergent adverse event, *US* United States

#### Analysis of adverse events by maximum CTCAE grade

When AEs were analyzed by maximum CTCAE grade, the percentage of patients who experienced at least 1 AE regardless of causality was similar in the two arms: 76/77 patients (98.7 %) in Arm A and 68/71 patients (95.8 %) in Arm B (absolute risk difference between the two arms: 0.029; *p* = 0.281, 95 % CI: -0.024, 0.082). The incidence of bone pain, febrile neutropenia, laryngeal hemorrhage, somnolence, and syncope was significantly lower in Arm A than Arm B (Table 4). The percentage of patients who experienced at least 1 AE possibly related to study drug was also similar in the two arms: 69/77 patients (89.6 %) in Arm A and 64/71 patients (90.1 %) in Arm B (absolute risk difference between the two arms: 0.005; *p* = 0.915, 95 % CI: -0.092, 0.103). The incidence of dysguesia and febrile neutropenia possibly related to study drug was significantly lower in Arm A than Arm B (Table 4).

**Table 4:** Summary of adverse events that significantly differed in incidence between arms by maximum CTCAE grade

Event	US commercial cetuximab Arm A (N = 77) n (%)	BI-manufactured cetuximab Arm B (N = 71) n (%)	Arm A vs Arm B *p* value (95 % CI)
*Regardless of causality*			
Bone pain	2 (2.6)	9 (12.7)	0.020 (0.016, 0.186)
Febrile neutropenia	1 (1.3)	11 (15.5)	0.002 (0.054, 0.230)
Laryngeal hemorrhage	0 (0.0)	6 (8.5)	0.010 (0.020, 0.149)
Somnolence	0 (0.0)	4 (5.6)	0.040 (0.003, 0.110)
Syncope	0 (0.0)	5 (7.0)	0.020 (0.011, 0.130)
*Possibly related to study drug*			
Dysgeusia	1 (1.3)	6 (8.5)	0.044 (0.002, 0.141)
Febrile neutropenia	1 (1.3)	8 (11.3)	0.012 (0.022, 0.177)

*Abbreviations*: *BI* Boehringer Ingelheim, *CI* confidence interval, *CTCAE* Common Terminology Criteria for Adverse Events, *US* United States

#### Adverse events of special interest for cetuximab treatment

There were no significant differences between Arm A and Arm B in the incidence of acneiform rash, cardiac events, infusion reactions, or hypomagnesemia (Table 5). A little over half of the patients in each arm reported acneiform rash possibly related to study drug, mostly grade 1 or 2 in severity. Grade 3 skin reactions possibly related to study drug were dermatitis acneiform (Arm A: 4/77 patients, 5.2 %; Arm B: 4/71 patients, 5.6 %) and rash (Arm A: no patients; Arm B: 3/71 patients, 4.2 %). Two patients in Arm A died of cardiac arrest; one death from cardiac arrest was considered possibly related to study drug. No grade 3 or 4 cardiac events were reported in Arm A and no grade 4 or 5 cardiac events were reported in Arm B. Few patients reported grade 3 or 4 infusion reactions (Table 6). In Arm A, 2 patients (2.6 %) experienced grade 4 anaphylactic shock and 1 patient (1.3 %) experienced a grade 4 infusion-related reaction; these events were possibly related to study drug. No grade 4 infusion reaction events were reported in Arm B. Approximately one-third of patients in each arm reported hypomagnesemia possibly related to study drug, mostly grade 1 or 2 in severity. Grade 3 hypomagnesemia possibly related to study drug occurred in 3/77 patients (3.9 %) in Arm A and 3/71 patients (4.2 %) in Arm B.

**Table 5:** Treatment-emergent adverse events of special interest for cetuximab

Adverse event	Relatedness of adverse event	US commercial cetuximab Arm A (N = 77) n (%)	BI-manufactured cetuximab Arm B (N = 71) n (%)	Arm A vs Arm B *p* value (95 % CI)
Acneiform rash^a^	Regardless of causality	42 (54.5)	40 (56.3)	0.826 (−0.142, 0.178)
	Possibly related to study drug	41 (53.2)	40 (56.3)	0.706 (−0.129, 0.191)
Cardiac event^b^	Regardless of causality	5 (6.5)	10 (14.1)	0.128 (−0.022, 0.174)
	Possibly related to study drug	2 (2.6)	6 (8.5)	0.120 (−0.015, 0.132)
Infusion reaction^c^	Regardless of causality	6 (7.8)	5 (7.0)	0.862 (−0.077, 0.092)
	Possibly related to study drug	5 (6.5)	4 (5.6)	0.826 (−0.068, 0.085)
Hypo-magnesemia	Regardless of causality	29 (37.7)	29 (40.8)	0.692 (−0.126, 0.189)
	Possibly related to study drug	26 (33.8)	23 (32.4)	0.859 (−0.138, 0.165)

^a^ Composite term of 16 MedDRA Preferred Terms including dermatitis acneiform and rash

^b^ Composite term of 39 MedDRA Preferred Terms including cardiac arrest and myocardial infarction

^c^ Composite term of 9 MedDRA Preferred Terms including infusion-related reaction and anaphylactic shock

*Abbreviations*: *BI* Boehringer Ingelheim, *CI* confidence interval, *MedDRA* Medical Dictionary for Regulatory Activities, *US* United States

**Table 6:** Grade 3/4 infusion reactions

Adverse event	Relatedness of adverse event	US commercial cetuximab Arm A (N = 77) n (%)	BI-manufactured cetuximab Arm B (N = 71) n (%)
Infusion-related reaction	Regardless of causality	1 (1.3)	1 (1.4)
	Possibly related to study drug	1 (1.3)	1 (1.4)
Anaphylactic shock	Regardless of causality	3 (3.9)	0 (0)
	Possibly related to study drug	3 (3.9)	0 (0)
Hypersensitivity reaction	Regardless of causality	0 (0)	1 (1.4)
	Possibly related to study drug	0 (0)	1 (1.4)
Pyrexia	Regardless of causality	0 (0)	0 (0)
	Possibly related to study drug	0 (0)	0 (0)

*Abbreviations*: *BI* Boehringer Ingelheim, *US* United States

#### Deaths

In Arm A, there were 11 deaths during the study, of which 3 deaths were considered possibly related to study drug (cardiac arrest, hemorrhage intracranial, and septic shock). In Arm B, there were 7 deaths during the study, of which 1 death was considered possibly related to study drug (septic shock).

### Efficacy

There were no clinically meaningful differences in efficacy between Arm A and Arm B; median OS, median PFS, ORR, and DCR were similar in the two arms (Table 7).

**Table 7:** Summary of efficacy outcomes

Outcome	US commercial cetuximab Arm A (N = 77)	BI-manufactured cetuximab Arm B (N = 71)	Unadjusted HR Arm A vs Arm B (95 % CI; *p* value)
OS (months), median (95 % CI)	9.23 (7.10, 11.80)	9.46 (6.87, 11.43)	0.92 (0.619, 1.368; *p* = 0.681)
PFS (months), median (95 % CI)	4.70 (3.52, 5.82)	5.65 (4.04, 6.47)	1.04 (0.717, 1.497; *p* = 0.850)
Overall response rate, % (95 % CI)	29.9 (19.6, 40.1)	36.6 (25.4, 47.8)	NA
Disease control rate, % (95 % CI)	58.4 (47.4, 69.4)	62.0 (50.7, 73.3)	NA
Complete response, n (%)	3 (3.9)	3 (4.2)	NA
Partial response, n (%)	20 (26.0)	23 (32.4)	NA
Stable disease, n (%)	22 (28.6 %)	18 (25.4 %)	NA

*Abbreviations*: *BI* Boehringer Ingelheim, *CI* confidence interval, *HR* hazard ratio, *NA* not applicable, *OS* overall survival, *PFS* progression-free survival, *US* United States

## Discussion

Overall, this study showed no clinically meaningful differences in safety between patients receiving US commercial cetuximab (Arm A) and those receiving BI-manufactured cetuximab (Arm B), using the same therapeutic regimen as the EXTREME study [3]. The combination of US commercial cetuximab with cisplatin or carboplatin plus 5-FU did not result in any unexpected safety signals in the treatment of patients with locoregionally recurrent and/or metastatic SCCHN. The AEs reported in this study are consistent with the known safety profile of cetuximab, cisplatin/carboplatin, or 5-FU, or with the underlying disease.

The safety profile of US commercial cetuximab (Arm A) was similar to that of BI-manufactured cetuximab (Arm B). All-grade, all-cause TEAEs with the highest incidences included nausea, fatigue, and hypomagnesemia in both arms. There were no AEs for which the incidence was significantly higher in patients receiving US commercial cetuximab than those receiving BI-manufactured cetuximab. Conversely, the incidence of rash, bone pain, febrile neutropenia, laryngeal hemorrhage, somnolence, syncope (regardless of causality), dysguesia, and febrile neutropenia (possibly related to study drug) was significantly lower in patients receiving US commercial cetuximab than those receiving BI-manufactured cetuximab. These between-group differences were not considered clinically meaningful. The safety profile of US commercial cetuximab in combination with chemotherapy in the current study is also consistent with that previously observed for BI-manufactured cetuximab in combination with the same chemotherapy regimen in the EXTREME study [3]. Commonly reported AEs (>10 %) in both studies included neutropenia, thrombocytopenia, and anemia, which are typically associated with cisplatin/carboplatin and 5-FU treatment [8–10]. In the current study, the incidence of febrile neutropenia (by maximum CTCAE grade and by grade 3/4) was 1.3 % in patients receiving US commercial cetuximab and 15.5 % in patients receiving BI-manufactured cetuximab. In the EXTREME study, the incidence of grade 3/4 febrile neutropenia was 5 % in both the cetuximab plus chemotherapy and chemotherapy-alone groups [3].

There were no significant differences in the incidence of AEs of special interest for cetuximab (acneiform rash, cardiac events, infusion reactions, and hypomagnesemia) [5] between patients receiving US commercial cetuximab and those receiving BI-manufactured cetuximab. As reported in other studies of cetuximab [3, 4, 11], the majority of skin reactions reported in both arms in the current study were grade 1 or 2 in severity. Overall, the frequency and nature of cardiac events, infusion reactions, and hypomagnesemia observed in patients receiving US commercial cetuximab in the current study are consistent with the known safety profile of cetuximab [5]. In the EXTREME study, the incidence of cardiac events was approximately 9 % in both the cetuximab plus chemotherapy group and the chemotherapy-alone group; the incidence of death attributed to cardiovascular death or sudden death was 3 % and 2 % in the cetuximab plus chemotherapy group and chemotherapy-alone group, respectively [5]. In the same study, the incidence of hypomagnesemia was 14 % and 6 % in the subset of patients receiving cetuximab plus cisplatin and 5-FU and the subset of patients receiving cisplatin and 5-FU alone, respectively; the incidence of grade 3/4 hypomagnesemia was 7 % and 2 %, respectively [5].

Compliance and study treatment exposure to cetuximab, as assessed by median duration of treatment and cumulative dose of cetuximab, were similar in the two arms. The relative dose intensity was more than 85 % for both US commercial cetuximab and BI-manufactured cetuximab during the combination chemotherapy period. The relative dose intensities of cisplatin, carboplatin, and 5-FU were also high in both treatment arms, ranging from around 80 % to 95 %, suggesting that the various combinations of agents used in this study were well tolerated. These results are consistent with the observation in the EXTREME study that the addition of cetuximab to cisplatin/carboplatin plus 5-FU did not affect the tolerability of the chemotherapy regimen [3]. In the EXTREME study, the median (interquartile range) duration of cisplatin and carboplatin treatment in the cetuximab arm was 15 weeks (6 to 19) and 18 weeks (10 to 19), respectively [3]. The relative dose intensity of cisplatin was ≥80 % in 89 % of patients and the relative dose intensity of carboplatin was ≥80 % in 93 % of patients in the cetuximab arm in the EXTREME study [3].

There were no clinically meaningful differences in OS, PFS, ORR, or DCR between patients receiving US commercial cetuximab and those receiving BI-manufactured cetuximab. While it should be noted that the current study was not designed or powered to assess efficacy, the efficacy results of this study are consistent with those reported in the EXTREME study. Median OS with US commercial cetuximab and BI-manufactured cetuximab was 9.23 months and 9.46 months, respectively, in the current study and 10.1 months with BI-manufactured cetuximab in the EXTREME study [3]. Median PFS with US commercial cetuximab and BI-manufactured cetuximab was 4.70 months and 5.65 months, respectively, in the current study and 5.6 months with BI-manufactured cetuximab in the EXTREME study [3]. The ORR with US commercial cetuximab and BI-manufactured cetuximab was 29.9 % and 36.6 %, respectively, in the current study and 36 % with BI-manufactured cetuximab in the EXTREME study.

A strength of the current study was analyzing the safety of two formulations of a drug in a randomized controlled trial. A limitation of the study was that the sample size was based on practical and clinical considerations. This was done to ensure that assessment of the safety profile could be appropriately compared between the two treatment arms, rather than on any statistical assumptions or hypotheses. However, the planned sample size was not met because the supply of BI-manufactured cetuximab under evaluation expired during the study. In addition, not all patients in Arm B received BI-manufactured cetuximab for the duration of the study: 9/71 patients in Arm B were switched to US commercial cetuximab when the supply of BI-manufactured cetuximab expired. The potential bias that this may have introduced was addressed by choosing the earliest date that a patient on Arm B was switched from BI-manufactured cetuximab to US commercial cetuximab as the cut-off date for the primary safety analysis. However, this early cut-off date also limited the duration of the safety period for this analysis.

## Conclusions

The safety profile of US commercial cetuximab in patients with locoregionally recurrent and/or metastatic SCCHN is consistent with the safety profile of BI-manufactured cetuximab in the current study and that reported in the previously published EXTREME study [3]. The combination of US commercial cetuximab with cisplatin or carboplatin plus 5-FU did not result in any unexpected safety signals; the AEs reported in this study are consistent with the known safety profile of cetuximab, cisplatin/carboplatin, or 5-FU, or with the underlying disease. In addition, there were no clinically meaningful differences in OS, PFS, or ORR between patients receiving US commercial cetuximab and those receiving BI-manufactured cetuximab. These results indicate that, despite potentially higher systemic exposures with US commercial cetuximab relative to BI-manufactured cetuximab, the safety profile of US commercial cetuximab is consistent with the safety profile of cetuximab in the current US prescribing information [5].

## Abbreviations

AE: adverse event
AUC: area under the concentration curve
BI: Boehringer Ingelheim
CI: confidence interval
CR: complete response
CTCAE: Common Terminology Criteria for Adverse Events
DCR: disease control rate
EGFR: epidermal growth factor receptor
EU: European Union
EXTREME: Erbitux in First-Line Treatment of Recurrent or Metastatic Head and Neck Cancer
FDA: Food and Drug Administration
5-FU: 5-fluorouracil
IVRS: interactive voice response system
KPS: Karnofsky Performance Status
MedDRA: Medical Dictionary for Regulatory Activities
ORR: overall response rate
OS: overall survival
PFS: progression-free survival
PR: partial response
RECIST: Response Evaluation Criteria in Solid Tumors
RT: randomized and treated
SCCHN: squamous cell carcinoma of the head and neck
TEAE: treatment-emergent adverse event
US: United States
